# Prevalence and concordance of oral and genital HPV in women positive for cervical HPV infection and in their sexual stable partners: An Italian screening study

**DOI:** 10.1371/journal.pone.0205574

**Published:** 2018-10-18

**Authors:** Gianguido Cossellu, Luigi Fedele, Bouabid Badaoui, Francesca Angiero, Giampietro Farronato, Ermelinda Monti, Carlo Antonio Liverani, Chiara Gorni, Sara Botti

**Affiliations:** 1 Department of Biomedical, Surgical and Dental Sciences, Università degli Studi di Milano, Fondazione IRCCS Ca’ Granda, Ospedale Maggiore Policlinico, Milan, Italy; 2 Dipartimento Donna - Bambino - Neonato, Centro di riferimento per la prevenzione, la diagnosi e la cura della patologia genitale HPV correlate, Fondazione IRCCS Ca’ Granda, Ospedale Maggiore Policlinico, Milan, Italy; 3 Laboratory of Biodiversity, Ecology and Genome; Mohamed V University, Rabat, Maroc; 4 Department of Medical Sciences and Diagnostic Integrated, S. Martino Hospital, University of Genoa, Italy; 5 Parco Tecnologico Padano - CERSA, Integrative Biology Group, Lodi, Italy; Universidade Estadual de Maringa, BRAZIL

## Abstract

**Objectives:**

This cross-sectional study aimed to evaluate the prevalence and type of oral HPV-infection in women with a cervical HPV-lesion and in the oral and genital mucosa of their male partners.

**Methods:**

The study group comprised 44 sexually-active women, 20–45 years with abnormal PAP smear, not more than 6 months prior to referral together with the male partners cohabiting in stable partnerships. A detailed questionnaire was administered concerning the HPV-related risk factors. Oral swabs, oral rinses, cervical swabs and urine samples were collected. HPV DNA was detected using two different polymerase chain reactions (PCRs): MY09-11 and FAP59-64. Positive samples were genotyped by Sanger sequencing and the INNO-LiPA HPV Genotyping Extra II probe assay. The association with risk factors was assessed by fitting a generalized model, using the General Linear Model function in the R-software; correlations were calculated between all data.

**Results:**

HPV was detected in 84% of Cervical Samples, in 24.3% of oral samples and in one urine sample. Only 27% of the HPV-positive results were identical with both PCR DNA assays.

8 male had oral HPV-positive samples different from women cervical samples. In one couple the urine-male sample had the same HPV present in the female-cervical sample. A significant association resulted between women/oral sex practices and men/n. of partners.

**Conclusions:**

This study reports that women (20.4%) with a diagnosis of cervical-HPV and their male partners (30,7%) are at high risk for subclinical oral HPV infection.

## Introduction

The incidence of head and neck cancer has changed dramatically over the last 30 years. Prior to that time, the commonest of these cancers, squamous cell carcinoma, generally occurred in older adults with a history of tobacco and alcohol use. While smoking-related head and neck cancers have been declining overall, squamous cell carcinoma of the oropharynx has been increasing in many developed countries, including Australia, Canada, Denmark, Japan, Norway, Sweden, Netherlands, and United Kingdom [1–5]. Today, patients diagnosed with head and neck squamous cell carcinoma (HNSCC) are more likely to be adults in their 40s and 50s who have never smoked or used other tobacco products. These changes are chiefly due to the human papillomavirus (HPV) [6].

Recent advances in molecular techniques have progressively identified novel HPV-types and sub-genomic HPV sequences [7] and more than 200 different HPV-types are now recognized by the International HPV Reference Center. HPV is the most common sexually transmitted infection (STI), making up over half of all new and existing STIs [8]. The majority of adults will become infected at some point in life, with no symptoms whatsoever; the body’s immune system generally eliminates the infection. In approximately 1% of adults, HPV-16, the type most commonly associated with head and neck cancer, is detectable in the saliva [9]. HPV is mainly known for its association with benign (non-cancerous) lesions, such as genital warts, and it has long been recognized in association with cervical cancer. Today, HPV is the most common cause of oropharyngeal cancer (OC), presenting different symptoms from HPV-negative cancers: neck mass, sore throat, and fewer than 15% of subjects report a visible oral lesion [10]. HPV-positive OC are 3 to 5 times more common among men than women. [1, 11]

In view of the association among HPV, cervix uteri, and OC, HPV-positive OC has been considered to be a sexually transmitted disease, and the HPV-positive cervix to act as a virus reservoir [12]. Other studies have found that a history of cervical dysplasia or abnormal PAP-smears in women increased the risk of HPV-positive oral cancer in their male partners [9, 13]. However, there is still no consensus on these matters, nor on the relationship between genital and oral HPV-infection.

In view of these inconsistent data, the present cross-sectional study aimed to evaluate the prevalence and type distribution of oral HPV-infection in Italian immunocompetent women with a known cervical HPV-related lesion (abnormal PAP-smear) and in the oral and genital mucosa of their monogamous male partners.

## Materials and methods

### Ethics statement

The study, performed between March 2015 and May 2016, was approved by the Ethics Committee of the *Fondazione IRCCS Ca’ Granda-OSPEDALE MAGGIORE POLICLINICO*,Milan (Prot. n. 2765). All participants agreed to participate in the research and signed their informed consent. All data were analyzed anonymously.

### Study population

The initial study group comprised 106 sexually-active adult women, aged 20–45 years, who had been referred to the U.O. Ostetricia e Ginecologia-Centro di riferimento per la prevenzione, la diagnosi e la cura della patologia genitale HPV-correlate, Fondazione IRCCS Cà-Granda Ospedale Maggiore Policlinico—Milano, after an abnormal PAP smear, not more than 6 months prior to referral, within a cervical screening program with routine clinical and cyto/histological examination, together with the male partners of some of these women, cohabiting in stable partnerships (S1 Table). Exclusions were: 7 women for drug use or illness (immunosuppresors/antibiotics for HIV/oral infection), 15 women for incomplete or inadequate samples; 40 women and 2 men refused to enter the study. The final study group comprised 44 women and 26 male partners. Participants received a free clinical examination of the oral cavity and were evaluated by a trained oral clinician for clinical examination of the oral mucosa and cytological sampling at the Department of Biomedical, Surgical and Dental Sciences, UOC Chirurgia Maxillo-Facciale e Odontostomatologia of the above hospital. A detailed questionnaire was administered, addressing sexual practices, socio-economic and health status. In particular it concerned personal information (gender, age, education, marital status, pregnancies); medical history (oral or cervical lesions, type of lesion, HPV vaccination status, other cervical infections); known cofactors associated with the risk of developing high-grade squamous intraepithelial lesions (smoking and drinking); sexual behavior (age of first sexual intercourse, number of partners in the last year, frequency of sexual intercourse, use of birth control, practice of oral sex). (S2 Table)

### Data and sample collection

Oral swabs, oral rinses and cervical swabs were collected from the women; oral swabs, oral rinses, and urine samples were collected from their partners. Cervical swabs were collected using a brush. Oral rinses were obtained by rinsing the mouth with 10-15ml of water and spitting the rinse into a tube. Oral swabs were collected both from the buccal mucosa and the surface of the tongue using a specific swab (ORAcollect-DNA kit; DNA-genotek Inc.). Urine samples were collected using conventional sterile tubes. All samples were immediately frozen at -20°C.

### DNA extraction

Papillomavirus DNA was extracted from four different matrices: cervical swab (CS), urine (U), oral swab (OS) and oral rinse (OR). DNA from OS was extracted using the prepIT DNA extraction kit (DNAGenotek), for CS, U and OR samples, the “NucleoSpin Virus, Viral RNA and DNA isolation” kit (Macherey-Nagel) was used. Briefly, prior to sample lyses, CS samples were incubated in 500ul of sterile PBS at room temperature for 1 hour; 200ul of PBS were then used for DNA extraction. U and OR samples were thawed overnight at 4°C, then 3 ml of sample were centrifuged at 13,000 xg for 10 min. The pellet was resuspended in 200ul of sterile PBS and the DNA extraction protocol was applied. The resulting DNA was stored at -20°C until testing.

### HPV detection and genotyping

**Fig 1:**
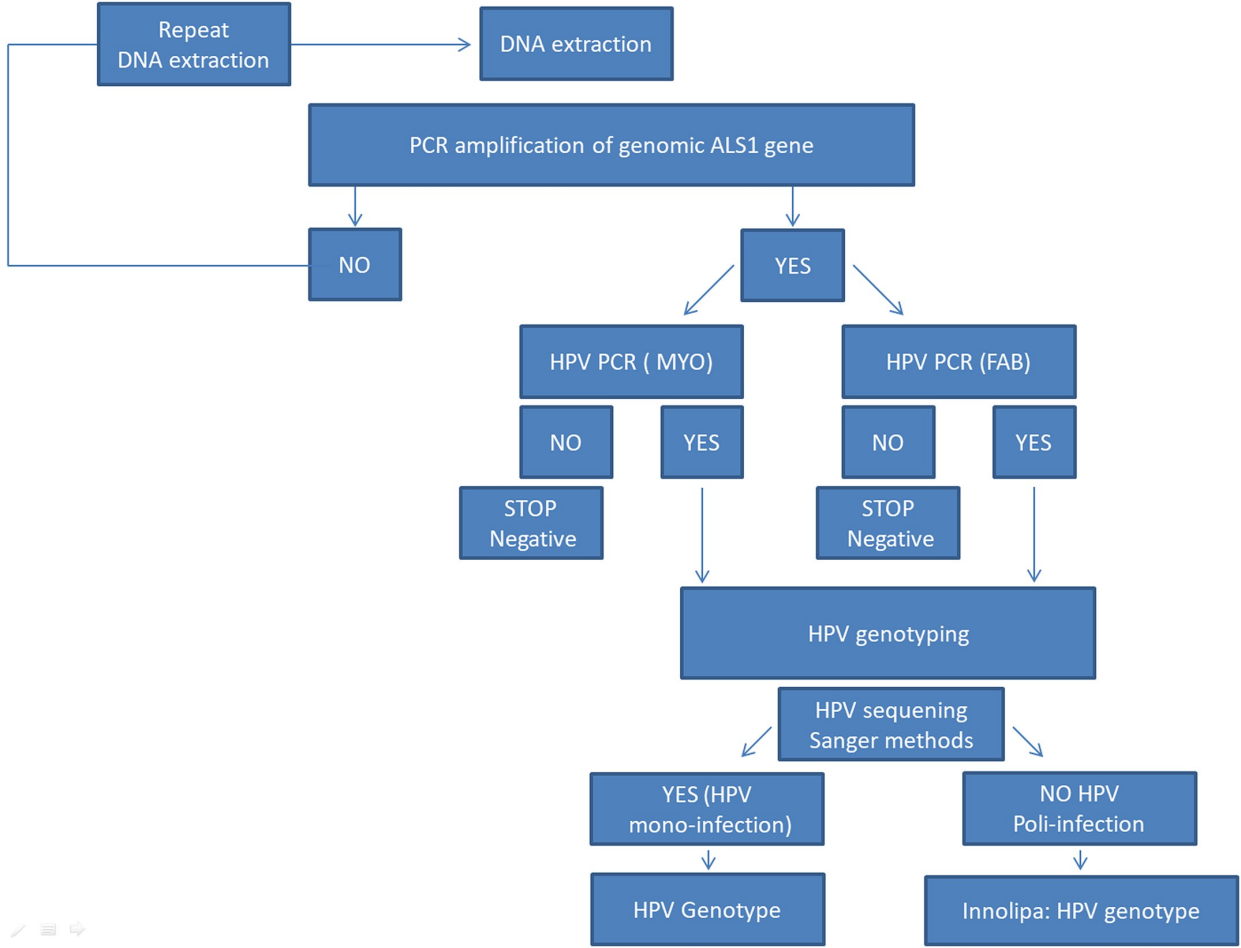
Molecular work flow.

The presence of human DNA in all samples was confirmed by amplifying the ALAS1 (5'-Aminolevulinate Synthase 1) gene (Bishop et al Genomics 1990). HPV DNA was detected using two different polymerase chain reactions (PCRs) targeting the HPV L1 region:i) MY09-11_HPV PCR assay and ii) FAP59-64_HPV PCR assays using respectively degenerate primers MY09 5'-GCMCAGGGWCATAATAATGG 3' MY11 5' CGTCCMARRGGAWACTGATC 3' to amplify a 450 bp fragment (Gravitt et al. 2000) and FAP59 5'TAACWGTIGGICAYCCWTATT 3') and FAP64 5'CCWATATCWVHCATITCICCATC-3' to amplify a fragment of about 480 bp (Forslund et al. 1999). The HPV DNA positive samples were subsequently genotyped by Sanger sequencing on the 3730xl DNA Analyzer (Applied Biosystems). Sequence traces from HPV DNA samples were aligned to the genomic sequence (PAVE Papilloma Virus Reference database or NCBI nucleotide database). The INNO-LiPA HPV Genotyping Extra II probe assay, based on the principle of reverse hybridization, was thus used to genotype the human HPV in samples with multiple HPV-infections. The following HPV genotypes can bedetected and identified: HPV 16, 18, 31,33, 35, 39, 45, 51, 52, 56, 58, 59, 68; 26, 53, 66, 70, 73, 82; 6, 11, 40, 42, 43, 44, 54, 61, 62, 67, 81, 83, 89. (Fig 1)

### Statistical analyses

Variables considered for statistical analyses were: gender, age, number of partners, oral sex, alcohol consumption per week, cervical lesions, pregnancy, vaccination, education, civil status, smoking, frequency of sexual intercourse, contraceptive use, other cervical lesions, age at first intercourse. Before analyzing the data, intervals were constructed for some variables: age, infection, alcohol consumption, and smoking. The effect of the variables on the HPV-infection status was assessed by fitting a generalized model, using the GLM (General Linear Model) function in the R software. The family “binomial” was used to describe the error distribution and link function. As the number of samples was relatively small, the R fisher.test function was used to perform Fisher’s exact test for verification. The model was tested using the maximum likelihood to evaluate the effect of inclusion of each parameter on the model. As supplementary analysis, the correlations were calculated between all parameters.

## Results

### Distribution of HPV infection in the study population (Table 1)

**Table 1:** Percentage of cervical swab (n. 43), oral swab (n. 70) or oral rinse (n.70) samples that were positive (P), doubtful (D) or negative (N) for the presence of HPV DNA using MY09-11 HPV, FAP59-64, or both HPV PCR assays.

	Cervical Swab (n. 43)			Oral Swab (n. 70)			Oral Rinse (n.70)		
	% P	% D	% N	% P	% D	% N	% P	% D	% N
MY09-11	81	0	19	18.6	2.9	78.5	10	0	90
FAP59-64	70	9	21	0	5,7	94.3	1	0	99
Both assays	84	0	16	20	2.9	78.5	10	0	90

Human genomic DNA corresponding to the ALAS1 (5'-Aminolevulinate Synthase) gene was detected in all samples (cervical swab, oral samples and urine). Only one cervical swab was inadequate.

#### Female—Cervical swab infection

HPV DNA was detected in 84% (n.36/43) of CS samples using the MY09-MY11_HPV DNA assay and/or the FAP59-FAP64_HPV DNA assay. The percentages of HPV-positive CS samples found with the two different PCR assays are in Table 1. Only 69.7% of the HPV PCR results were identical (positive/positive; negative/negative) with the two assays while 16% of the results were discordant (e.g. positive/negative; doubtful/positive; doubtful/negative) and 14% were concordant but not identical (e.g. weakly positive/positive). S3 Table

#### Male and female—Oral sample infection (OR+OS)

HPV DNA was detected in 24.3% of oral samples. An oral sample was considered positive if HPV DNA was detected in OS or OR using the MY09-11_HPV and/or the FAP59-64_HPV PCR assays (S3 Table). In particular HPV DNA was present in 20.4% of women with a diagnosis of cervical-HPV and 30.7% of male. Moreover HPV DNA was present in 18,6% of OS and in 10% of OR (male+female) using the MY09-11 (see Table 1). Considering the OS samples, only 75.6% of the HPV DNA results were identical with the two assays; 18.6% of results were discordant.**1.3 Male—Urine samples**.

HPV DNA was detected in only one sample (S3 Table).

### Genotyping

#### Female—Cervical swab samples

**Fig 2:**
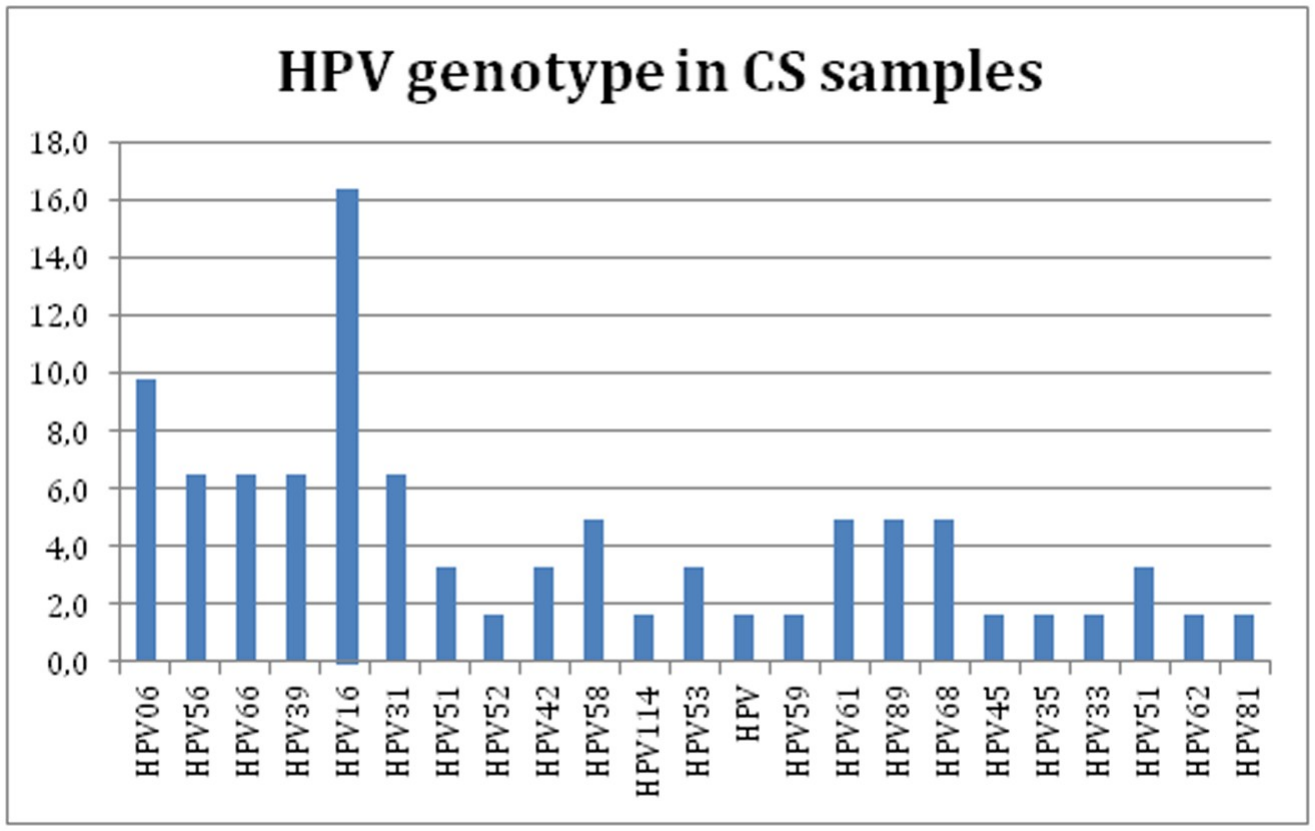
Prevalence of HPV genotype in CS samples using MY09-MY11, FAP59-FAP64, or INNO-LiPA HPV Genotyping Extra II probe assays; HPV genotype (X-axis) and percentage of HPV genotype (y-axis).

All positive HPV DNA CS samples (Fig 2) were genotyped by sequencing. Only 27% of the HPV genotyping results were clearly identical with both PCR DNA assays (e.g. HPV type 16 with both assays). In some cases the HPV genotyping results were different (e.g. HPV type 33 amplified with MY09-11 PCR assay and HPV type 68 with FAP59-64 assay). In other cases only using one of both PCR assays was possible characterize the HPV type in a sample (S3 Table). The percentage of multiple infections was 18% with the MY09-11 PCR assay and 35% with the FAP59-64 assay, and was 45% if both assays were considered. The INNO-LiPA HPV Genotyping Extra II probe assay was used in samples infected with multiple HPV types. HPV16 was the most frequent HPV type in CS samples.

#### Male and female—Oral samples (OR+OS)

**Fig 3:**
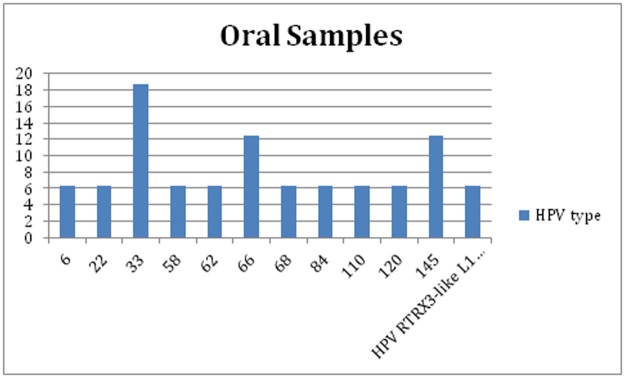
HPV genotyping: Percentage of HPV type in oral samples using MY09-MY11, FAP59-FAP64, or INNO-LiPA HPV Genotyping Extra II probe assays.

All positive HPV DNA oral samples were genotyped by sequencing. The sequences obtained using the MY09-11 PCR assay gave more complete results than those obtained using the FAP59-64 PCR assay: only one sample that was positive using the FAP59-64 PCR DNA assay was genotyped (HPV type 147). All oral samples were genotyped using the MY09-11 PCR assay (Fig 3). There were no oral samples with multiple infections, with one exception: HPV147 with the FAP59-FAP64 PCR DNA assay and HPV type 33 with the MY09-MY11 PCR assay. HPV33 was the most frequent HPV type in oral samples.

### HPV concordance in women (both in cervix and oral cavity, Table 2)

**Table 2:** HPV type in women with both oral and cervical swab (CS) samples positive for HPV.

Woman patient	Oral Tot Genotype	Cervical Swab (CS) Genotype
10	HPV33	HPV control 1; HPV control 2; HPV16; HPV31; HPV51;HPV52
33	HPV-RTRX3-like L1 gene	HPV control 1; HPV control 2
39	HPV62	HPV59
41	HPV22	HPV control 1; HPV61; HPV89
55	HPV6	HPV6
68	HPV145	HPV control 1; HPV control 2; HPV16; HPV31; HPV39; HPV06; HPV66; HPV62; HPV81
72	58	42

16% (7/43) of the oral samples from CS-HPV-positive women were positive. The HPV genotype in the oral sample was different from that in the CS sample for patients no. 10,39 and 72. The same HPV06 was detected in patient 55. The HPV-types in patients 33, 41 and 68 are unfortunately not included in the list of HPV-types detected by the INNO-LiPA HPV Genotyping Extra II probe assay.

### HPV transmission between partners: Inter-transmission (Table 3)

**Table 3:** HPV data from couples with the woman’s cervical swab positive for HPV and the man’s oral samples or urine samples positive for HPV.

No. of couples	Cervical Swab HPV DNA (female)	Cervical Swab HPV Type (female)	Oral HPV DNA (male)	Oral HPV Type sanger Sequencing (male)	Urine HPV DNA (male)
3	Pos	39	pos	68	neg
4	Pos	HPV control 1; HPV control 2; HPV16; HPV31; HPV51;HPV52	pos	66	neg
5	Pos	16	pos	33/147	neg
15	Pos	HPV control 1; HPV61; HPV89	neg	n.a.	pos*
17	Pos	68	pos	110	neg
18	Pos	66	pos	120	neg
19	Pos	HPV control 1; HPV6	pos	33	neg
22	Pos	HPV control 1; HPV51	pos	84	neg
24	Pos	43/52	pos	66	neg

*HPV type in US: HPV control 1; HPV control 2; HPV58;HPV61

A total of 25 couples were analyzed. 21 women had HPV-positive CS; 4 had HPV-negative CS and their partners had HPV-negative oral samples.

9 male partners had HPV-positive samples (8 Oral samples and 1 US). In couple no 15, the male had US with the same HPV61-type present in the CS sample. In the other couples, the HPV type in the CS sample differed from that in the male sample.

In couple no. 22, the male OS was positive for HPV84, which unfortunately is not on the list of HPV-types detected by the INNO-LiPA HPV Genotyping Extra II probe assay.

### Statistical analysis

The regression of variables against total infection status highlighted three significant factors: gender, oral sex, and alcohol (p = 0.000224, 0.0119, and 0.0362, respectively). This shows that there is a male-female difference with regard to HPV-infection; oral sex is determinant for HPV-infection; alcohol consumption appears to have a negative effect on HPV-infection status. Other variables, such as number of partners, frequency of intercourse, age, or use of birth control, do not emerge as significant in this study.

In particular, the regression of these variables against oral infection status shows that gender is a significant factor (p = 0.04517). Age and smoking came close to significance (p = 0.0885 and 0.0901, respectively). Oral sex and alcohol emerged as significant factors also for cervical infections (p = 0.0492 and 0.0229, respectively) while number of partners, use of birth control, smoking, and age appear not to affect cervical infections.

In a second phase of the study, the data were subdivided into male and female samples. Two regressions were then performed using the two data-subsets. It emerged that oral sex is highly significant in women for HPV-infection, but only indicatively significant in men. Oral sex was three times more common in men than in women. Moreover, in men the number of the partners and the frequency of intercourse tend to affect oral HPV-infection (p = 0.0514 and 0.08, respectively: approaching significance). As the sample is relatively small, the significance of oral sex and alcohol was checked and confirmed using the Fisher exact test. Correlations were also calculated between all parameters, leading to some important biological considerations. Particularly, the correlation between oral sex and cervical infection was significant (p = 0.04001).

## Discussion

The diagnosis of cervical HPV-infection is not generally accompanied by investigations at different sites, such as the oral cavity, except in the presence of visible lesions. The present results show that patients with genital HPV-infection are at risk for oral infection, not always associated with visible clinical lesions. The absence of clinical signs in the oral cavity suggests the infection may be subclinical, and a molecular assay might thus be necessary to diagnose it. Mixed HPV genotype infections are common, and there are 182 different Homo sapiens HPV types in the Papilloma virus Genome Database (PaVE: https://pave.niaid.nih.gov/). Due to the great variability of HPV types, two different HPV PCR assays were used, to amplify all genotypes simultaneously and Sanger sequencing to identify all 182 types present in the HPV database, and also SNP variants between the same HPV-types (e.g. the SNP variant between different HPV 16 types). If the same HPV 16 subtype is present in the partner, a direct mechanism of HPV transmission may be suggested. Moreover, SNP comparison is important to clarify the genetic evolution of types over time, their carcinogenic properties, and to differentiate re-infection from persistent infection [14]. Although Sanger technique is highly accurate, samples with infection by multiple HPV-types cannot be genotyped without cloning so the INNO-LiPA HPV Genotyping assay was for samples with multiple HPV-infections. However, this method can only detect 32 HPV types, and does not detect SNP variability.

It is now known that HPV is present in up to 60% oropharyngeal squamous cell cancer (OSCC), and a rise of HPV-associated to OSCC has recently been detected. The peculiarity of this increase is that it runs counter to the decline in smoking, which was considered one of the main risk factor associated with the development of this type of cancer.[15,16]

These trends are confirmed for the USA,[17] where HPV was detected in 16% of tonsillar cancer samples between 1984 and 1989, against 73% between 2000 and 2014, as well as in Europe[18], specifically Sweden, where it rose from 23% in the 1970s to 77% in 2003.

Research worldwide has attempted to elucidate HPV transmission rates to the oropharynx and has recently focused upon the possible transmission from the cervix to a woman’s oral mucosa and that of her partner (Table 4).

**Table 4:** Concurrence of oropharyngeal HPV-screening studies. Summary of the transmission and concordance of HPV from cervix to oropharynx reported in the literature.

Year	References	Country	Women with genital HPV lesions	Male Partner	F Oral + (%)	M Oral + (%)	Oral sampling method	Oral Genotypes	Notes/risk factors	Method
2016	Uken [39]	Germany	101	60	3	5	Brush	35, 41, 61, 84–16, 18	Low transmission	PCR
2016	Visalli [38]	Italy	100	-	24	-	Saliva collection	16, 18, 45, 31	High risk cervical lesions	PCR
2016	Kero [37]	Finland	58	58	10,3	24,1	Brush	16, 6, 53, 56	Low genotype concordance—Smoking, oral sex practice	PCR
2015	Tatar[36]	Hungary	40	34	20	17,6	Brush	16		PCR
2015	Marques [35]	Brazil	43	22	2,3	13,6	Brush	-		PCR
2014	Vidotti [34]	Brazil	105	-	23,8	-	Brush	-	Genital infection	PCR
2014	Meyer [33]	Germany	70	5	5,7	0	Brush+Rinse	54, 84	Low transmission	NestedPCR
2014	Fuster-Rossello [32]	Argentina	30	-	30	-	Brush	16, 52, 6		PCR
2013	Adamopoulou [31]	Greece	29	-	17,2 58,6*	-	Rinse	16, 31, 53, 73, 6, 77, 81, 84		PCR and Nested PCR
2011	Peixoto [30]	Brazil	100	-	81	-	Brush	-	Alcohol	PCR
2011	Termine [29]	Italy	98	-	14,3	-	Rinse	16, 3, 10, 107	Younger age sexual debut	Nested PCR
2010	Saini [28]	Malaysia	70	-	5,71	-	Brush		Only 13 HR hpv tested	PCR
2010	Sanchez-Vargas [27]	Mexico	46	-	100	-	Brush	16	-	PCR
2009	Termine [26]	Italy	140	-	1,4	-	Brush	-	Rinse better?	Nested PCR
2009	Castro[25]	Brazil	30	-	0	-	Brush	-	-	PCR
2008	Marais [24]	South Africa	72	-	23,6	-	Brush	11, 28, 33, 68, 72	Low concordance	Nested PCR
2006	Giraldo [23]	Brazil	70	-	37,1	-	Brush	-	Genital infection	PCR
2006	Fakhry [22]	USA	35	-	14,3	-	Rinse	45, 83, 89, 72, 71, 62	Genital infection	PCR
2004	Smith [21]	USA	165	-	3,6	-	Rinse	16, 31	-	PCR
1998	Badaracco [20]	Italy	10	-	50	-	Brush	-	Only 10 subjects	PCR
1992	Kellokoski [19]	Finland	309	-	3,8	-	Brush	6, 11, 16	-	HY

The main limit of these studies is that authors haven’t designed a research protocol as we did considering both female and their stable partners, collecting different type of samples, the use of different analyses technique and the association with other risk factors.

21 studies published between 1992 and 2017 have collected data from various countries[19–39]. Subgroups are here considered, based on macro-geographic areas: 10 from Europe, 7 South America, 2 North America, 1 South Africa, 1 Asia/Malaysia. Four of the European studies were conducted in Italy[20,26,29,38]. Only 5 recent articles (4 in Europe and 1 in Brazil) analyzed the oral mucosa of both female and male partners[35–39].

The pooled prevalence obtained from Table 4 (all countries) of oral HPV-infection in women with cervical HPV-infection is 18.3%. In Europe, 955 women with HPV cervical infection have been evaluated, of whom 95 were positive (10%); in South America, of 424 women 188 were positive (44.3%). It appears to be important to consider different geographical areas separately, due to the different results that might be associated with different socio-economic and behavioral lifestyles.

With regard to the male samples, the difference is not significant: positivity is 14.6% in Europe and 13.6% in South America. These data should be carefully interpreted due to the small sample size and limited number of studies (22 subjects in South America, 157 in Europe).

With regard to Italian studies, the pooled prevalence among a total cohort of 348 women with cervical HPV-infection was 45 positive in the oral cavity. Among HPV-negative women in Italy, the concurrence of HPV in both genital and oral mucosa was 12.9%[20,26,29,38]. The positivity rate for women found here (20.4%) was higher than the world average (18.3%), the European average (10%) and other Italian studies (12.9%).

No other Italian study investigated the presence of the virus in the oral mucosa of male partners. As far as the present authors are aware, only 5 previous studies investigated HPV transmission from the female cervix to the sexual partner’s oral mucosa. The present study may be considered to be the first Italian study to investigate the presence of the virus in the oral mucosa of stable male partners of women with genital infection. Thus these preliminary results may only be compared with those from other countries (4 in Europe and 1 in Brazil), which reveal an overall cumulative rate of 14.5%, compared with 30.7% reported here.

The association with different risk factors (alcohol consumption, smoking, no. of partners, etc.) and other factors (use of condom, oral sex etc.) was examined in this study (S1 Table). For women, the results suggest a significant association with oral sex practices and for men with increased number of partners. Study participants also underwent a complete oral clinical examination, but no relevant clinical lesions were found; thus oral examination alone cannot exclude the possibility of oral HPV-infection. Oral HPV-infection was mainly sub-clinical.

Transmission of HPV to the oral cavity, and the consequent risk of oral cancer, is increased in women with cervical infection and in their partners, suggesting the possible transmission between oral cavity and genitals. The presence of the virus in these two anatomic sites could be consequent upon genetic predisposition, conditions of low/altered immune response, the host’s inability to develop an appropriate immune response. However, the viral concordance between the two anatomical sites appears not to be mandatory.

Possible explanations for the different prevalence of HPV genotypes in the oropharynx versus the cervix might include different clearance mechanisms, host defense, or local cellular factors (lysozymes, amylase, lactoferrin, immunoglobulin a, etc.).

The presence of the virus in the genital mucosa of male partners’ urine samples was also investigated. Only one positive sample was found (genotype 22); this was the same genotype found in his female partner but no assumption may be based on a single case.

The main limits of this study are the small sample size and the absence of a control group; these are two of the principal critical points common to other published studies. However, some comparison with the healthy population may emerge from recent studies of children and adults (women without cervical HPV-infection), that report oral prevalence of HPV ranging between 2.5 and 9%[11, 29, 40].

The strengths of this research are the use of highly efficient and sensitive procedures for oral sample collection (rinse+dedicated brush); the HPV detection with two different PCR assays (FAP59-64 and MY09-11); the genotyping processing techniques used (Sanger+InnoLipa); a molecular analyses able to detect each single SNP variant; the evaluation of the compresence of the virus in stable couples with cervical HPV-infection; a complete questionnaire including all the main risk factors associated with HPV-infection; a review of the previous articles published. Further, this is one of the few studies that included the male partners of women with cervical HPV-infection, and the first such Italian sample.

## Conclusion

This study reports that women with a prior diagnosis of cervical HPV and their male partners are at high risk for subclinical oral HPV, as indicated by the presence of the virus in the oral cavity in 20.4% of female and in 30,7% of male. The pooled prevalence of oral HPV-infection in women with cervical HPV-infection obtained from a review of the literature 1990–2016 was 10% (European average) and 12.9% in Italian studies. This is significantly higher than that reported in the general population of women without cervical HPV-infection.

The same trend emerged in male samples: pooled published data gives a positivity of 14.5%, whereas the present study found double this rate (30.7%). This might indicate that genital HPV-infection is a predisposing condition to oral HPV-infection, in both women and their male partners. However, the results should be interpreted cautiously, and larger cohorts must be tested via multicentre and longitudinal studies to clarify viral transmission to/from the oral mucosa.

## Supporting information

S1 Table Age and genital lesion/PAP test results of the study group. (XLSX)

S2 Table Complete sociodemographic characteristics of the study group. (XLSX)

S3 Table Results from cervical, oral and urina samples with the different type of PCR DNA assays. (XLSX)
